# Expression patterns of members of the ethylene signaling–related gene families in response to dehydration stresses in cassava

**DOI:** 10.1371/journal.pone.0177621

**Published:** 2017-05-23

**Authors:** Meng Yun Ren, Ren Jun Feng, Hou Rui Shi, Li Fang Lu, Tian Yan Yun, Ming Peng, Xiao Guan, Heng Zhang, Jing Yi Wang, Xi Yan Zhang, Cheng Liang Li, Yan Jun Chen, Peng He, Yin Dong Zhang, Jiang Hui Xie

**Affiliations:** 1 College of Agronomy, Hainan University, Haikou, P.R. China; 2 Key Laboratory of Biology and Genetic Resources of Tropical Crops, Ministry of Agriculture, Institute of Tropical Bioscience and Biotechnology, Chinese Academy of Tropical Agricultural Sciences (CATAS), Haikou, P.R. China; 3 Hainan Institute of Science & Technology, Haikou, P.R. China; 4 Chinese Research Academy of Environmental Sciences, Beijing, P.R. China; 5 Ministry of Agriculture Key Laboratory for Rubber Biology, Rubber Research Institute, Chinese Academy of Tropical Agricultural Sciences, Danzhou, Hainan, P.R. China; National Taiwan University, TAIWAN

## Abstract

Drought is the one of the most important environment stresses that restricts crop yield worldwide. Cassava (*Manihot esculenta* Crantz) is an important food and energy crop that has many desirable traits such as drought, heat and low nutrients tolerance. However, the mechanisms underlying drought tolerance in cassava are unclear. Ethylene signaling pathway, from the upstream receptors to the downstream transcription factors, plays important roles in environmental stress responses during plant growth and development. In this study, we used bioinformatics approaches to identify and characterize candidate *Manihot esculenta* ethylene receptor genes and transcription factor genes. Using computational methods, we localized these genes on cassava chromosomes, constructed phylogenetic trees and identified stress-responsive cis-elements within their 5’ upstream regions. Additionally, we measured the trehalose and proline contents in cassava fresh leaves after drought, osmotic, and salt stress treatments, and then it was found that the regulation patterns of contents of proline and trehalose in response to various dehydration stresses were differential, or even the opposite, which shows that plant may take different coping strategies to deal with different stresses, when stresses come. Furthermore, expression profiles of these genes in different organs and tissues under non-stress and abiotic stress were investigated through quantitative real-time PCR (qRT-PCR) analyses in cassava. Expression profiles exhibited clear differences among different tissues under non-stress and various dehydration stress conditions. We found that the leaf and tuberous root tissues had the greatest and least responses, respectively, to drought stress through the ethylene signaling pathway in cassava. Moreover, tuber and root tissues had the greatest and least reponses to osmotic and salt stresses through ethylene signaling in cassava, respectively. These results show that these plant tissues had differential expression levels of genes involved in ethylene signaling in response to the stresses tested. Moreover, after several gene duplication events, the spatiotemporally differential expression pattern of homologous genes in response to abiotic and biotic stresses may imply their functional diversity as a mechanism for adapting to the environment. Our data provide a framework for further research on the molecular mechanisms of cassava resistance to drought stress and provide a foundation for breeding drought-resistant new cultivars.

## Introduction

Drought is the one of the major environmental stresses which reduce yield and constrain crop production. In some areas, where suboptimal rainfall occurs frequently, it is important to enhance plant resistance or tolerance to drought in order to minimize losses. Cassava (*Manihot esculenta* Crantz) is a perennial crop native to tropical America [1, 2] that is widely cultivated for its tuberous roots (starchy storage roots) and is a staple food for humans and animals [3]. The high starch content makes cassava an excellent candidate for energy production [4–6]. Currently, cassava ranks sixth in terms of global crop production. Cassava shares many traits in common with other tropical crops, such as high drought and heat tolerance, and reduced requirement for agricultural fertilizers. However, the mechanisms underlying drought tolerance in cassava remain unclear.

The gaseous hormone ethylene is an endogenous regulator involved in a wide variety of plant growth and developmental processes including seed germination, responses to abiotic and biotic stresses, fruit ripening, senescence of leaves and flowers, sex determination and abscission [7, 8]. Ethylene biosynthesis and signal transduction pathways have been widely investigated in the model plant Arabidopsis (*Arabidopsis thaliana*) [9]. It is mediated by a group of five ethylene receptors ((ETR1 (ethylene receptor 1), ERS1 (ethylene response sensor 1), ERS2, ETR2, and EIN4 (ethylene-insensitive 4)) that bind ethylene and promote downstream responses [9–11]. The receptors are primarily localized to the endoplasmic reticulum [12]. Ethylene receptor genes have been identified in many plant species, and their expression is regulated in a manner dependent on tissue type, developmental stage, environmental cues as well as during fruit ripening [13–15].

In ethylene signaling pathway, EIN3/EIL and ERF are two primary transcription factors, which are necessary and sufficient for the induction of the vast majority of ethylene response genes [16, 17]. As the requisite component for ethylene signaling [18], EIN3/EIL is a nuclear protein downstream of EIN2 [19] that is positively regulated by EIN2 [20]. EIN3/EIL activates ERF1 transcription by binding to a specific sequence in the promoter of ERF1 which acts downstream of EIN3/EIL. [21] ERF proteins are a subfamily of the APETALA2 (AP2)/ ethylene-responsive-element-binding protein (EREBP) transcription factor family that are unique to plants [22]. To date, ERFs have been identified in many plant species, including Arabidopsis [23], rice (*Oryza sativa*) [23], tomato (*Lycopersicon esculentum*) [24], soybean (*Glycine max*) [25], and wheat (*Triticum aestivum*) [26]. The large size of the ERF family encompasses abiotic and biotic stress responses [27–31]. Although genes involved in ethylene transduction pathways play important roles in plant responses to stresses, they have not been fully investigated in cassava.

In this study, we measured expression levels of a subset of candidate ethylene receptor genes (*MeETR2*, *MeERS1*, *MeERS2*, *MeERS3*, *MeERS4*, *MeERS5* and *MeEIN4*) and transcription factor genes (*MeEIL1*, *MeEIL2*, *MeERF1*, *MeERF2*, *MeERF3*, *MeERF4*, *MeERF5*, *MeERF6*, *MeERF7*, *MeERF8*, *MeERF9*, *MeERF10*, *MeERF11*, *MeERF12*, *MeERF13* and *MeERF14*) in cassava SC5 after treatments with drought, polyethylene glycol 6000 (PEG-6000) or NaCl, aiming to identify changes in their expressional profiles among control, drought, osmotic and salt stress conditions. This study provides a theoretical basis for further exploring the molecular mechanism of cassava drought resistance, and lays the foundation for cultivating new drought resistant varieties and breeding materials.

## 2 Materials and methods

### 2.1 Plant material, growth conditions and stress treatments

A local cassava cultivar (SC5) was obtained from the Institute of Tropical Bioscience and Biotechnology, Chinese Academy of Tropical Agricultural Sciences, China, in 2013. Plants were grown in plastic flower pots (23 cm in height, 26 cm in diameter and 16 cm in bottom diameter) filled with a mixture of vermiculite and sand (1:1 V/V), with a small amount of coconut fiber. The abiotic treatment experiments are carried out after plantlets were grown for 3 months. To obtain optimum concentrations for osmotic and salt stresses, 5%, 10%, 20%, 30%, 40%, and 50% (w/v) PEG-6000 and 50 mM, 100 mM, 200 mM, 300 mM, 400 mM, and 500 mM NaCl were applied to potted cassava for 3 days in preliminary experiments. According to these results, severe stresses (400 mM NaCl and 30% PEG-6000) were used in subsequent experiments. As mentioned above, three month samples were collected at 0, 1, 2, 4, 8, 12, and 24 h after treatment with 30% PEG-6000 or 400 mM NaCl. For drought treatment, water was withheld from the plants for 18 days and tissues were collected at 0, 3, 6, 9, 12, 15, and 18 d timepoints. For well-watered controls, plants were regularly watered every three days. The experiments were carried out with three independent biological replicates.

After the experiment treatments, plants were removed carefully from the plastic flower pots. Root, tuberous root, stem, and leaf tissues were collected from cassava plants, and all the samples were immediately frozen in liquid nitrogen and stored at −80°C until use.

### 2.2. Identification and isolation of ethylene receptor genes and transcription factor genes

To isolate ethylene receptor and transcription factor genes in cassava, the sequences of Arabidopsis ethylene receptors and transcription factors were retrieved from The Arabidopsis Information Resource (TAIR; http://www.arabidopsis.org; Tair10). The sequences of ethylene receptors and transcription factors in cassava were searched by BLAST using the sequences of Arabidopsis ethylene receptors and transcription factors as the query at Phytozome (http://phytozome.jgi.doe.gov/pz/portal.html). BLAST protocols and database search parameters were as follows: database, Phytozome v11.0.8; species, *Manihot esculenta* v6.1 (the cultivar AM560-2); search type, BLAST; target type, genome; program, TBLASTX; expect threshold, -5; comparison matrix, blosum62; word length, 3; allow gaps; and filter query.

The cDNA sequences of likely ethylene receptor and transcription factor genes in the cassava cultivar SC5 were cloned, sequenced, and then aligned with those of the cultivar AM560-2. Identification was considered positive if the sequences of these genes had more than 95% homology to their counterparts from the cassava cultivar AM560-2. Identified genes were re-annotated and named.

### 2.3. Gene positions on cassava chromosomes

Chromosomal locations of cassava ethylene receptor and transcription factor genes were obtained using the BLAT server and additional physical localization tools available with the cassava genome database (http://phytozome.jgi.doe.gov/pz/portal.html). BLAT protocols and database search parameters were as follows: database, Phytozome v11.0.8; species, *Manihot esculenta* v6.1(the cultivar AM560-2); Minimum # of matches, 2; Minimum score, 30; Minimum identity (%), 95; Maximum gap, 2; Tile Size, 11; Maximum intron size, 750000.

### 2.4. Cis-elements within promoter sequences

The 1.5 kb genomic sequence located upstream of the ATG start codon of the 5’-UTR of the ethylene receptor and transcription factor genes representing their core promoter region were obtained from the cassava genome database (http://phytozome.jgi.doe.gov/pz/portal.html). An upstream cis-element analysis was performed using PlantCare online software (http://bioinformatics.psb.ugent.be/webtools/plantcare/html/).

### 2.5. Phylogenetic analysis

To generate a phylogenetic tree, the predicted amino acid sequences of ethylene receptors and transcription factors of Arabidopsis and Cassava were aligned using the ClustalX program (version 1.83) [32]. Phylogenetic and molecular evolutionary analyses were conducted using MEGA 5.0 program with the neighbor-joining (NJ) method [33]. Reliability of the obtained trees was tested using bootstrapping with 1,000 iterations [34].

### 2.6. qRT-PCR analysis

Total RNA was extracted from cassava tissues using the CTAB method [35]. DNase I treatment was carried out using RNase-free DNase I (Thermo Scientific, USA). RNA concentration and purity was quantified for each sample using UV-Vis Spectrophotometer (UVP, USA). The first-strand cDNA synthesis was performed using 1 μg of total RNA from each sample using a cDNA Synthesis Kit (Thermo Scientific, USA) according to the manufacturer’s instructions. After reverse transcription, the products of each reaction were diluted 10-fold in sterile water to avoid potential primer interference in the subsequent qRT-PCR reactions. qRT-PCR was carried out using primer pairs (S1 Table) designed by Applied Biosystems Primer Express software. A sample of diluted cDNA was subjected to qRT-PCR in a final volume of 20μl containing 10μl SYBR Select Master Mix (Life Technologies, USA) and gene specific primers (0.2 uM). qRT-PCRs were performed on a real-time PCR machine (STRATAGENE, MX3500) using the following cycling parameters: an initial denaturation for 2 min at 95°C, followed by 40 cycles of 15 s at 95°C and 1 min at 58–62°C (varying with specific primer pairs whose amplification efficiencies were between 0.9 and 1.1), and a final cycle of 1 min at 95°C, 30 s at 55°C and 30 s at 95°C to obtain the melting curve. To normalize for the total amount of cDNA present in each reaction, *MeActin* (Manes.12G150500.1/ cassava4.1_033108m.g) gene was co-amplified as an endogenous control for calibration of relative expression [36]. The threshold cycle (Ct) values of target and *MeActin* genes were obtained through qRT-PCR. The relative expression for all selected genes was quantified using the 2^-ΔΔCt^ method, where ΔΔCt = (Ct _target gene_ − Ct _*MeActin* gene_) _treatment_ − (Ct _target gene_ − Ct _*MeActin* gene_) _control_ [37]. Three biological replicates and three technical replicates were analyzed for qRT-PCR. Expressional data were calculated as log_2_-based values and divided by the control, and were then used for heat map generation based on MeV software. Genes with log_2_ (relative expression values)-absolute values > 1 and *p* value < 0.05 (determined by two-tailed Student's *t*-test) were considered as being significantly regulated.

### 2.7. Quantitation analysis of trehalose and proline

Measurements of physiological indicators of stress were obtained using commercial kits, trehalose assay kit (Cat. No. SY6) and proline assay kit (Cat. No. JY4) (Suzhou Comin Biotechnology Co. Ltd., China). The trehalose content of cassava fresh leaves was measured using the anthrone-sulfuric acid colorimetric assay, whereby trehalose is generated into furfural in concentrated sulfuric acid under higher temperature, and the subsequent furfural reaction with anthrone forms a blue-green complex with maximum absorbance at 620 nm [38]. Proline was extracted with sulfosalicylic acid, and following a heat treatment, the proline and acidic-ninhydrin reaction generated a red substrate measured by absorbance at 520 nm [39]. Statistical analysis was verified by performing Student’s *t* -test (*p* value < 0.05 was considered statistically significant).

## 3. Results

### 3.1. Sequence alignments and phylogenetic analysis

**Fig 1:**
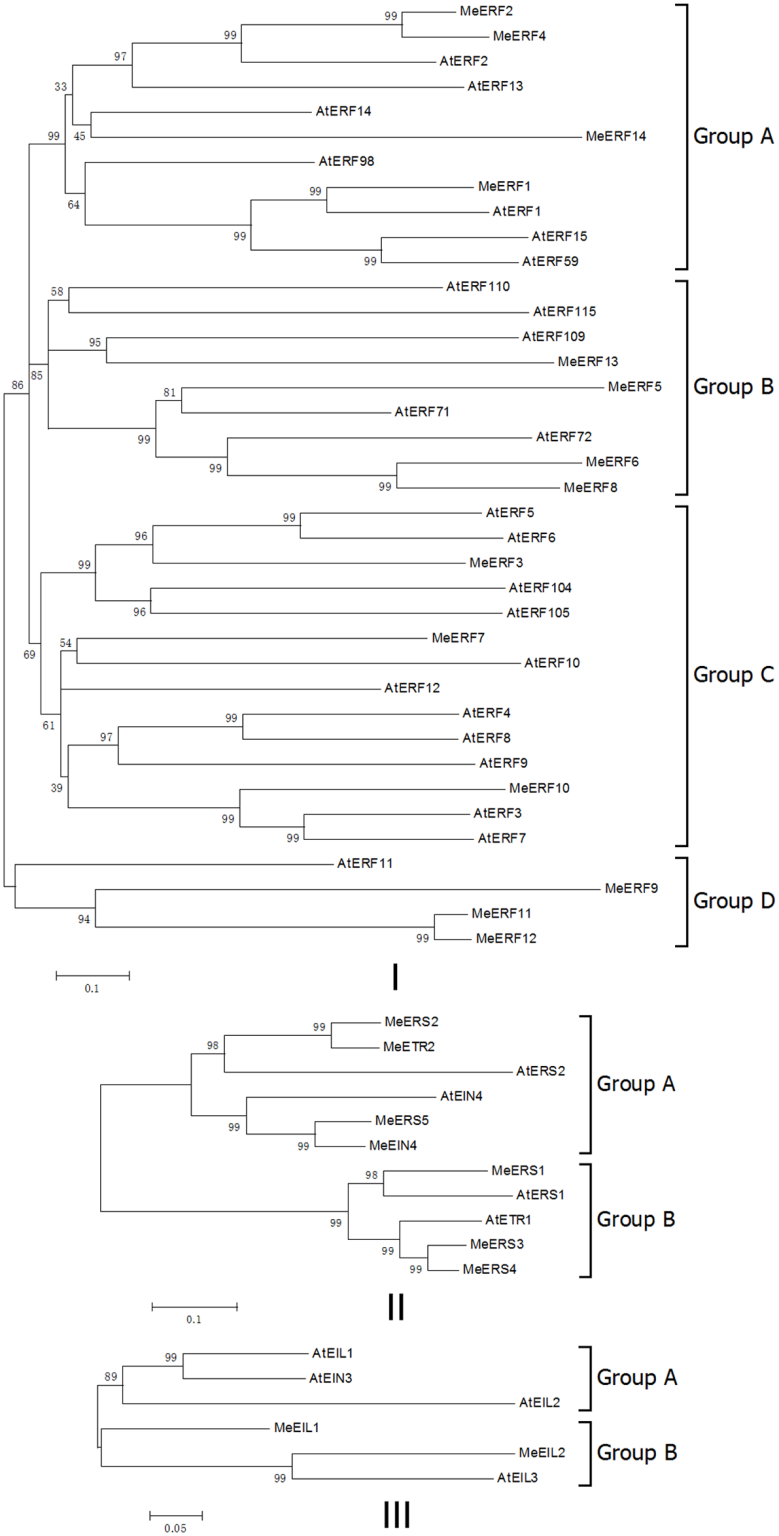
Phylogenetic trees of ethylene receptors and transcription factors in cassava (*Manihot esculenta*, Me) and Arabidopsis (*Arabidopsis thaliana*, At). The phylogenetic trees were generated using MEGA5.0 program with the neighbor-joining method. Bootstrap values from 1000 replicates are indicated at each branch. I, phylogenetic tree of ERFs that clustered into four distinct groups (A, B, C, and D groups); II, phylogenetic tree of ethylene receptors that clustered into two distinct groups (A and B groups); III, phylogenetic tree of EILs that clustered into two distinct groups (A and B groups).

To examine the phylogenetic relationships among ethylene receptors and transcription factors and group them within the established subfamilies, we constructed a phylogenetic tree from alignments of their amino acid sequences in cassava and Arabidopsis (Fig 1 and S2 Table). The phylogenetic tree (Fig 1-I) indicated that fourteen MeERFs could be divided into four groups (A, B, C, and D). Four cassava ERFs were classified into group B, together with five AtERFs from Arabidopsis, which suggested that despite the very recent duplication events, no significant change was happened for MeERFs in group B. Furthermore, MeERFs in group D experienced a significant expansion, as three MeERFs were present in cassava, while one corresponding ortholog existed in Arabidopsis. But a significant diminution of MeERFs was happened in group A and group C, since group A contained four MeERFs and seven AtERFs, and group C contained three MeERFs and eleven AtERFs. Among fourteen MeERFs, MeERF2 and MeERF4, MeERF6 and MeERF8, and MeERF11 and MeERF12 showed high sequence similarity with each other (Fig 1-I). The phylogenetic tree (Fig 1-II) showed that seven ethylene receptors from cassava fell into two groups (A and B), against with four ethylene receptors from Arabidopsis. Four ethylene receptors from cassava against with two corresponding orthologs from Arabidopsis in group A, so cassava ethylene receptors went through a significant expansion. Furthermore, three nodes (MeERS5/MeEIN4, MeERS2/MeETR2, and MeERS3/MeERS4) with high bootstrap confidence (≥ 99%) were identified in phylognetic analysis (Fig 1-II). The phylogenetic analysis (Fig 1-III) indicated that there were no cassava orthologs of AtEIL1, AtEIL2 and AtEIN3 from Arabidopsis in group A, and two MeEILs (MeEIL1 and MeEIL2) were present in cassava, while one corresponding ortholog existed in Arabidopsis in group B (Fig 1-III).

### 3.2 Chromosomal distribution of ethylene receptor genes and transcription factor genes

**Fig 2:**
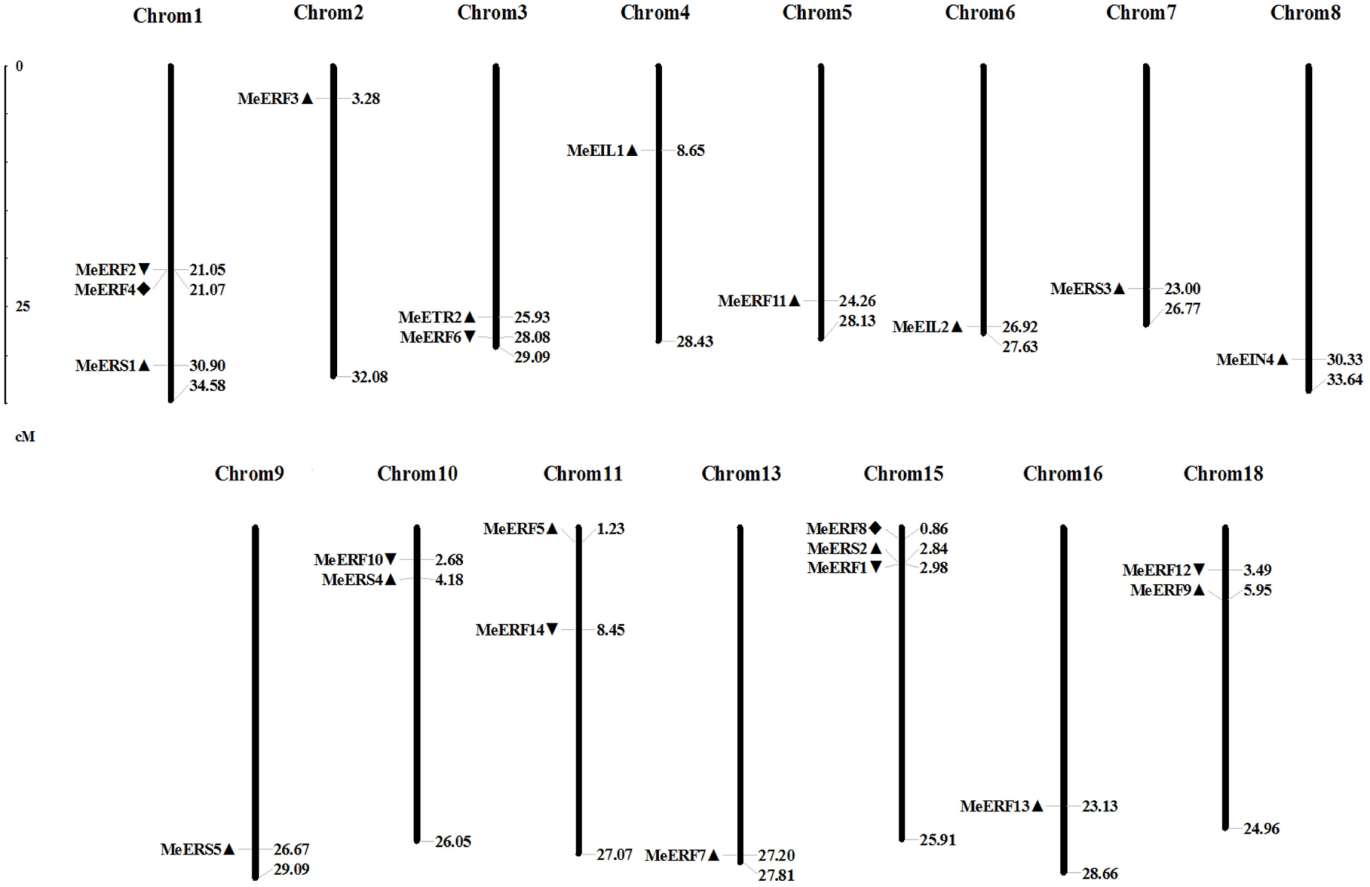
Distribution of ethylene receptor and transcription factor genes on cassava chromosomes. The chromosome numbers are indicated at the top of each bar. The gene names are on the left side of each chromosome according to the approximate physical locations (right side) of these genes. The triangles indicate the direction of transcription.

The locations of all ethylene receptor genes and transcription factor genes in the cassava genome were identified after analysis of chromosomal locations of these genes using the BLAT server and additional physical localization tools provided with the cassava genome database. These genes were anchored on fifteen of the 18 cassava chromosomes as shown in Fig 2. Multiple ethylene receptor genes and transcription factor genes were found on chromosome 3, 10, 11, 18 (two genes), and 1 and 15 (three genes), while only one was found on any of the other chromosomes. MeERF2/MeERF4 and MeERF9/MeERF12 genes diplaying very high similarity in the phylogenetic trees (Fig 1) were also located close to each other in the genome (Fig 2), which would indicate that recent tandem duplication had happened.

### 3.3. Cis-elements in promoter sequences of ethylene receptor genes and transcription factor genes

To further explore transcriptional regulation and the potential function of ethylene receptor genes and transcription factor genes, the cis-elements in their promoter sequences were predicted using the PlantCare database (S3 Table). Promoters of most of ethylene receptor genes and transcription factor genes contained the Skn-1 motif required for endosperm expression [40]. *MeERF2*, *MeERF11* and *MeERF12* had C-repeat/DRE regulatory elements involved in cold- and dehydration-responsiveness [41]. *MeERS1*/ *MeERF5*/ *MeERF9* promoters contained the MSA-like cis-regulatory element involved in cell cycle regulation [42]. Most of the promoters studied, with the exception of *MeEIL2*, *MeERF2* and *MeERF9*, contained cis-regulatory HSE elements involved in heat stress responses [43]. MBS (MYB binding sites) involved in drought inducibility, TC-rich repeats and other elements involved in defense and stress responsiveness were found in most of the promoters. The LTR, a low-temperature responsive cis-element, was found in *MeERS4*, *MeERF1* and *MeERF12*. Interestingly, most of these genes contained one or more cis-elements involved in signaling pathways involving ethylene, abscisic acid (ABA), salicylic acid (SA), jasmonic acid (JA), auxin and gibberellic acid. The cis-elements presenting in promoter regions of ethylene receptor genes and transcription factor genes suggested they might play an important role in cassava growth and development, and also in mediating responses to biotic and abiotic stresses.

### 3.4 Tissue specific expression of ethylene receptor genes and transcription factor genes

**Fig 3:**
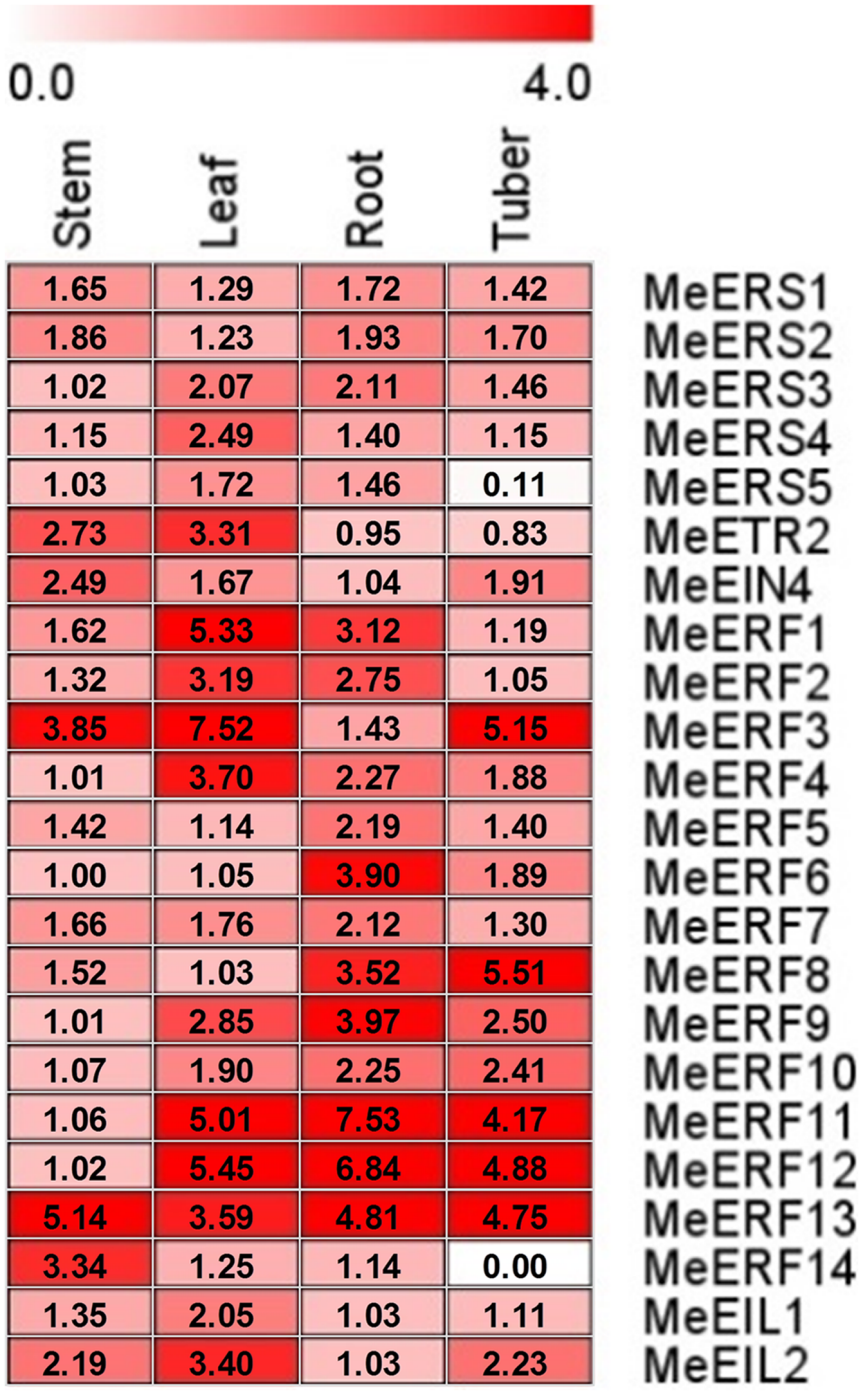
Expression profiles of ethylene receptor genes and transcription factor genes in different cassava tissues (stem, leaf, root, and tuber). The threshold cycle (Ct) values of target and *MeActin* genes were obtained through qRT-PCR. The expression values for all selected genes was quantified using the 2^–ΔCt^ method, where ΔCt = Ct _target gene_ − Ct _actin gene_ [37]. The Ct of the formula represent mean Ct values of three repeats from three independent qRT-PCR assays. The heat map was generated based on the log_2_ (expression values+1) with MeV software. Red indicates high expression and white indicates low expression.

Several ethylene receptor genes and transcription factor genes have been investigated with respect to their differential expression patterns in various plant tissues and organs [44, 45]. In this study, the expression of candidate ethylene receptor genes and transcription factor genes was profiled in different cassava organs by qRT-PCR (Fig 3, S4–S6 Tables). The relative mRNA levels demonstrated that the transcripts of most of the genes studied could be detected in all cassava tissues (stem, leaf, root, and tuber). However, expression of *ERF14* was not detected in tuberous root. The changes in expression of *MeERS1*, *MeERS2*, *MeERF5*, *MeERF7*, *MeERF13*, and *MeEIL1* were not significant in different tissues, and *MeERF13* was the only gene whose expression was more abundant in all cassava tissues. *MeEIN4* and *MeERF14* in stem and *MeERS4*, *MeERS5*, *MeETR2*, *MeERF1*, *MeERF2*, *MeERF3*, *MeERF4*, and *MeEIL2* in leaf had highest expression levels, except for *MeERS3*, *MeERF6*, *MeERF9*, *MeERF11*, and *MeERF12*, which exhibited highest expression in root and *MeERF8* and *MeERF10*, which had highest expression in tubers (Fig 3 and S4–S6 Tables).

### 3.5 Expression profiles of ethylene receptor genes and transcription factor genes in response to dehydration stresses in cassava leaves

**Fig 4:**
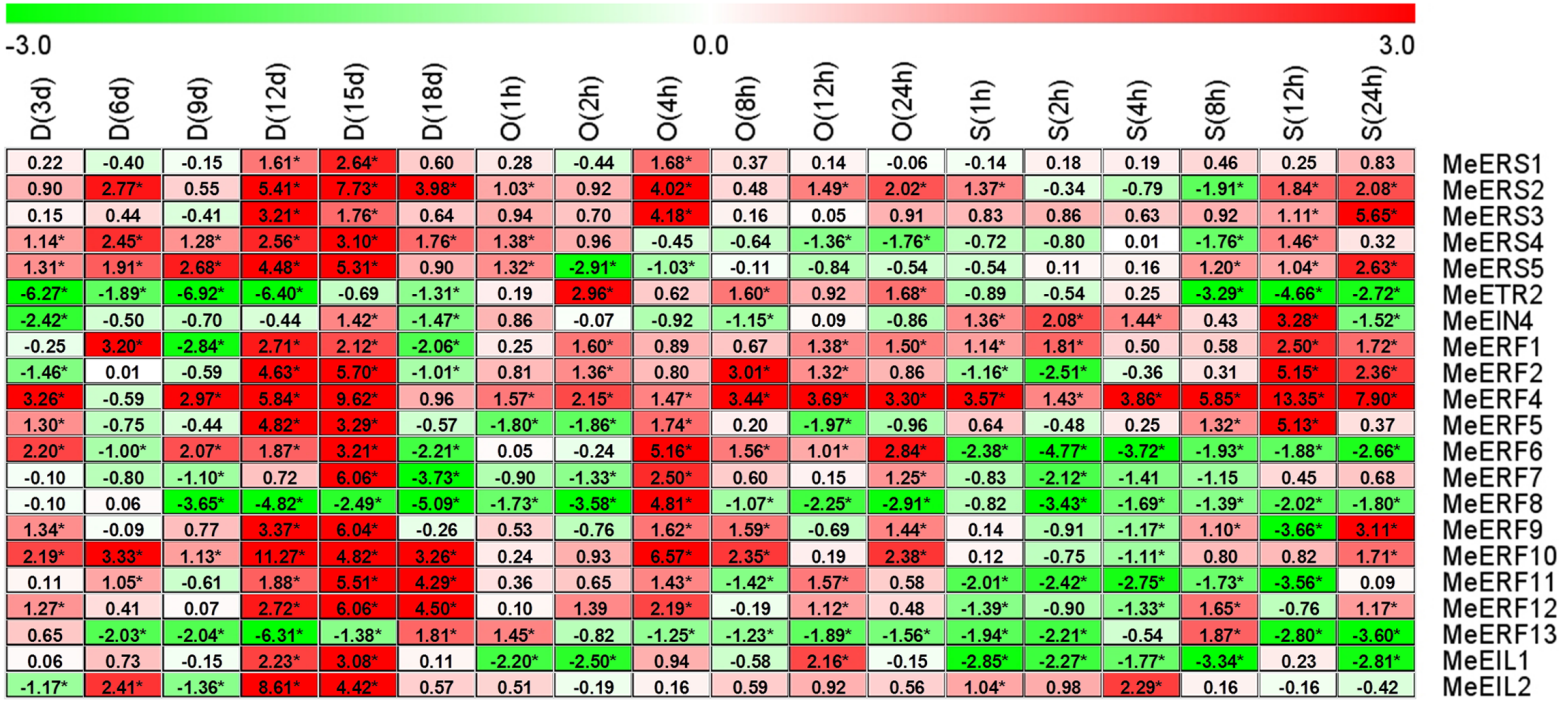
Expression profiles of ethylene receptor genes in cassava leaves after drought, osmotic, or salt treatment. The threshold cycle (Ct) values of target and *MeActin* genes were obtained through qRT-PCR. The relative expression values for all selected genes was quantified using the 2^-ΔΔCt^ method, where ΔΔCt = (Ct _target gene_ − Ct _*MeActin* gene_) _treatment_ − (Ct _target gene_ − Ct _*MeActin* gene_) _control_ [37]. Ct of the formula represent mean Ct values of three repeats from three independent qRT-PCR assays. The heat map was generated based on the log_2_ (relative expression values) with MeV software. Red indicates high expression and white indicates low expression. D, drought stress; O, osmotic stress; S, salt stress. Asterisk (*) on the right corner of number indicate a significant difference at *P* < 0.05 (Student’s *t*-test) and absolute log_2_ (relative expression values) > 1 compared with non-treated control.

In cassava leaf tissue, all of the tested ethylene receptor genes and transcription factor genes showed differential expression patterns in response to drought as well as osmotic and salt stresses, with the exception of *MeERF3* and *MeERF14*, which had transcript levels that were too low to detect (Fig 4, S7–S10 Tables). *MeERS2* and *MeERF1* at 83.33% of the time points, *MeERS3* and *MeERF4* at 94.44% of the time points, and *MeERF10* at 88.89% of the time points were induced in expression by drought, osmotic and salt stresses, but *MeERF8* at 88.89% of the time points was suppressed by the three stresses (Fig 4). In general, the number of induced ethylene receptor genes and transcription factor genes were clearly more than the number that were suppressed under all stress conditions in cassava leaves (36.51% vs. 22.49%, respectively; S10 Table). Drought stress induced the highest number of ethylene receptor genes and transcription factor genes in terms of elevated expression (47.62%) in cassava leaves, followed by osmotic stress (34.13%), salt stress (27.78%, S10 Table). But, salt stress suppressed the highest number of ethylene receptor genes and transcription factor genes in terms of reduced expression in leaves (30.95%), followed by drought stress (19.84%) and osmotic stress (16.67%, S10 Table).

The transcript levels of *MeERS1*, *MeERS2*, *MeERS3*, *MeERS4*, *MeERS5*, *MeERF1*, *MeERF2*, *MeERF4*, *MeERF5*, *MeERF6*, *MeERF9*, *MeERF10*, *MeERF11*, *MeERF12*, *MeEIL1*, and *MeEIL2* were significantly up-regulated or unchanged compared to the control at 6 time points after drought stress treatment in cassava leaves, except for *MeERF1* (9 d and 18 d), *MeERF2* (3 d and 18 d), *MeERF6* (6 d and 18 d), and *MeEIL2* (3 d and 9 d) which were significantly suppressed at only two time points. *MeETR2*, *MeEIN4*, *MeERF7*, *MeERF8*, and *MeERF13* were significantly down-regulated or unchanged at 6 time points after drought stress treatment in cassava leaves, except for *MeEIN4* (15 d), *MeERF7* (15 d), and *MeERF13* (18 d), which were significantly up-regulated at one time point after drought stress treatment (Fig 4).

*MeERS1*, *MeERS2*, *MeERS3*, *MeETR2*, *MeERF1*, *MeERF2*, *MeERF4*, *MeERF6*, *MeERF7*, *MeERF9*, *MeERF10*, *MeERF11*, *MeERF12*, and *MeEIL2* were significantly induced or maintained at a level similar to the control at all time points after osmotic stress treatment in cassava leaves, except for *MeERF7* (2 h) and *MeERF11* (8 h), which were only suppressed at one time point. *MeERS4*, *MeERS5*, *MeEIN4*, *MeERF5*, *MeERF8*, *MeERF13*, and *MeEIL1* were significantly down-regulated or unchanged at all time points after osmotic stress treatment in cassava leaves, except for *MeERS4* (1 h), *MeERS5* (1 h), *MeERF5* (4 h), *MeERF8* (4 h), *MeERF13* (1 h), and *MeEIL1* (12 h) which were significantly induced at one time point (Fig 4).

*MeERS1*, *MeERS2*, *MeERS3*, *MeERS5*, *MeEIN4*, *MeERF1*, *MeERF2*, *MeERF4*, *MeERF5*, *MeERF10*, and *MeEIL2* had significantly high or unchanged transcript levels compared to the control at all time points after salt stress treatment in cassava leaves, except for *MeERS2* (8 h), *MeEIN4* (24 h), *MeERF2* (1 h and 2 h), and *MeERF10* (4 h), which they were significantly reduced at one or two time points. But *MeERS4*, *MeETR2*, *MeERF6*, *MeERF7*, *MeERF8*, *MeERF9*, *MeERF11*, *MeERF12*, *MeERF13*, and *MeEIL1* had significantly low or unchanged transcript levels at all time points after salt stress treatment in cassava leaves, except for *MeERS4* (12 h), *MeERF9* (8 h and 24 h), *MeERF12* (8 h and 24 h), and *MeERF13* (8 h), which they were significantly increased at one or two time points (Fig 4).

### 3.6 Expression profiles of ethylene receptor genes and transcription factor genes in response to dehydration stresses in cassava stems

**Fig 5:**
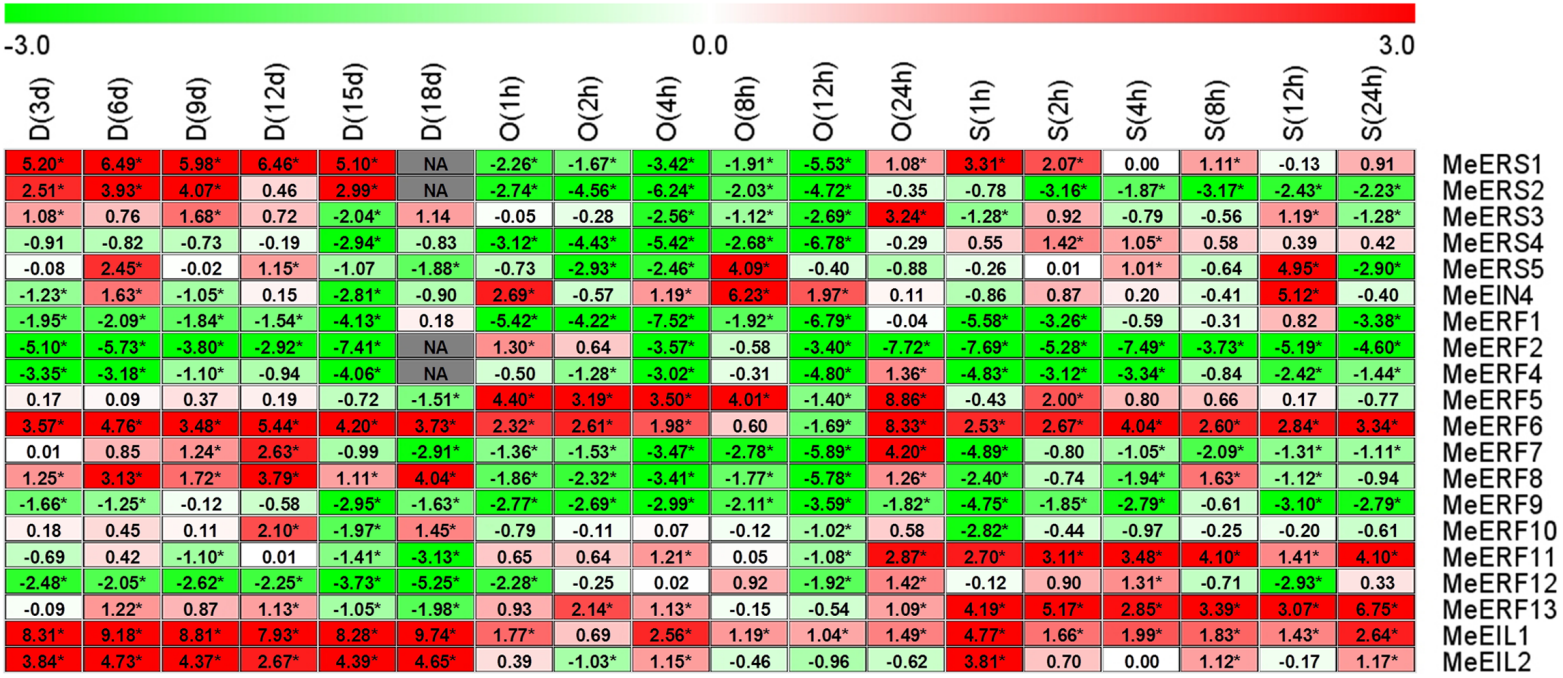
Expression profiles of ethylene receptor genes in cassava stems after drought, osmotic, or salt treatment. The threshold cycle (Ct) values of target and *MeActin* genes were obtained through qRT-PCR. The relative expression values for all selected genes was quantified using the 2^-ΔΔCt^ method, where ΔΔCt = (Ct _target gene_ − Ct _*MeActin* gene_) _treatment_ − (Ct _target gene_ − Ct _*MeActin* gene_) _control_ [37]. Ct of the formula represent mean Ct values of three repeats from three independent qRT-PCR assays. The heat map was generated based on the log_2_ (relative expression values) with MeV software. Red indicates high expression and white indicates low expression. D, drought stress; O, osmotic stress; S, salt stress. Asterisk (*) on the right corner of number indicate a significant difference at *P* < 0.05 (Student’s *t*-test) and absolute log_2_ (relative expression values) > 1 compared with non-treated control. NA denote the expression was too weak to detect.

In stem tissue, all of the tested ethylene receptor genes and transcription factor genes showed differential expression patterns in response to drought, osmotic, and salt stresses, with the exception of some genes that had transcript levels too low to detect, including *MeETR2*, *MeERF3*, and *MeERF14* at all time points in response to all stresses and *MeERS1*, *MeERS2*, *MeERF2*, and *MeERF4* at one time point (18 d) after drought stress treatment (Fig 5, S7, S11–S13 Tables). *MeERF6* at 94.44% of the time points and *MeEIL1* at 100% of the time points were induced in expression by drought, osmotic and salt stresses, but *MeERF1* at 88.89% of the time points, *MeERF2* at 88.24% of the time points, *MeERF4* at 94.12% of the time points and *MeERF9* at 100% of the time pointswere suppressed by the three stresses (Fig 5). In general, the number of ethylene receptor genes and transcription factor genes that were induced under all stress conditions was slightly less than the number that were suppressed in cassava stems (32.30% vs. 36.24%, respectively; S13 Table). Drought stress significantly induced the greatest number of ethylene receptor genes and transcription factor genes in terms of expression (37.93%) in cassava stems, followed by salt stress (32.50%) and osmotic stress (26.67%, S13 Table). The ranking for transcriptional suppression was osmotic stress (45.00%), drought stress (32.76%), and salt stress (30.83%, S13 Table).

*MeERS1*, *MeERS2*, *MeERS3*, *MeERS5*, *MeERF6*, *MeERF7*, *MeERF8*, *MeERF10*, *MeERF13*, *MeEIL1*, and *MeEIL2* levels were significantly elevated or unchanged compared to the control at all time points after drought stress, except for one or two time points where *MeERS3* (15 d), *MeERS5* (18d), *MeERF7* (18 d), *MeERF10* (15 d), and *MeERF13* (15 d and 18 d) were significantly reduced. But *MeERS4*, *MeEIN4*, *MeERF1*, *MeERF2*, *MeERF4*, *MeERF5*, *MeERF9*, *MeERF11*, and *MeERF12* were significantly reduced or unchanged throughout drought treatment in cassava stems, except for *MeEIN4* a significant increase at 6 d (Fig 5).

After osmotic stress treatment, *MeEIN4*, *MeERF5*, *MeERF6*, *MeERF11*, *MeERF13*, and *MeEIL1* were significantly up-regulated or maintained at a level similar to the control at all time points, except for 12 h where *MeERF5*, *MeERF6*, and *MeERF11* were significantly reduced. *MeERS1*, *MeERS2*, *MeERS3*, *MeERS4*, *MeERS5*, *MeERF1*, *MeERF2*, *MeERF4*, *MeERF7*, *MeERF8*, *MeERF9*, *MeERF10*, *MeERF12*, and *MeEIL2* were significantly down-regulated or maintained at a level similar to the control at all time points after osmotic stress treatment in cassava stems, except at one time point where *MeERS1* (24 h), *MeERS3* (24 h), *MeERS5* (8 h), *MeERF2* (1 h), *MeERF4* (24 h), *MeERF7* (24 h), *MeERF8* (24 h), *MeERF12* (24 h), and *MeEIL2* (4 h) were significantly increased (Fig 5).

For the salt stress treatment, *MeERS1*, *MeERS4*, *MeEIN4*, *MeERF5*, *MeERF6*, *MeERF11*, *MeERF13*, *MeEIL1*, and *MeEIL2* levels were significantly increased or unchanged compared to the control in cassava stems, but *MeERS2*, *MeERS3*, *MeERF1*, *MeERF2*, *MeERF4*, *MeERF7*, *MeERF8*, *MeERF9*, and *MeERF10* were significantly suppressed, except for *MeERS3* (12 h) and *MeERF8* (8 h) which were significantly up-regulated at one time point (Fig 5).

### 3.7 Expression profiles of ethylene receptor genes and transcription factor genes in response to dehydration stresses in cassava roots

**Fig 6:**
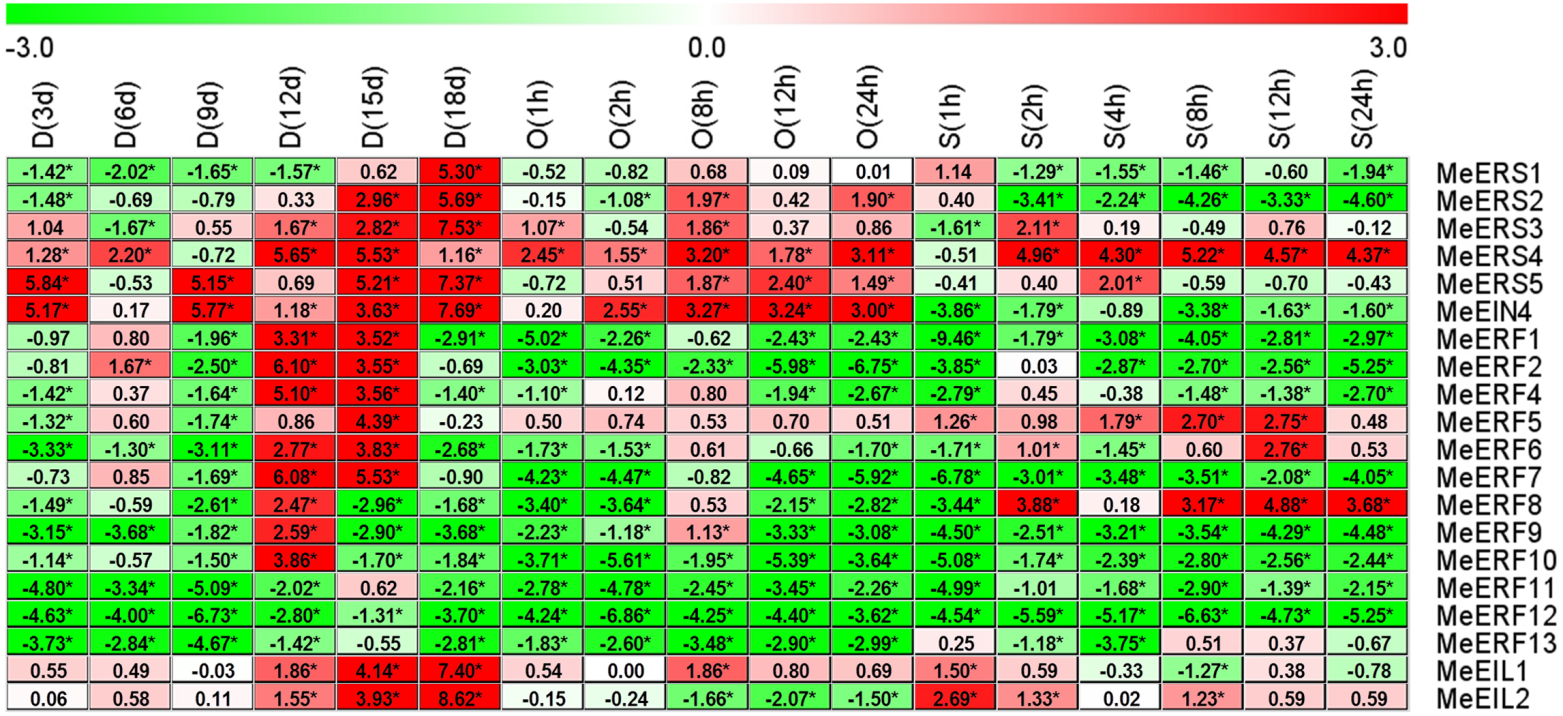
Expression profiles of ethylene receptor genes in cassava roots after drought, osmotic, or salt treatment. The threshold cycle (Ct) values of target and *MeActin* genes were obtained through qRT-PCR. The relative expression values for all selected genes was quantified using the 2^-ΔΔCt^ method, where ΔΔCt = (Ct _target gene_ − Ct _*MeActin* gene_) _treatment_ − (Ct _target gene_ − Ct _*MeActin* gene_) _control_ [37]. Ct of the formula represent mean Ct values of three repeats from three independent qRT-PCR assays. The heat map was generated based on the log_2_ (relative expression values) with MeV software. Red indicates high expression and white indicates low expression. D, drought stress; O, osmotic stress; S, salt stress. Asterisk (*) on the right corner of number indicate a significant difference at P < 0.05 (Student’s t-test) and absolute log_2_ (relative expression values) > 1 compared with non-treated control.

In root tissue, all of the tested ethylene receptor genes and transcription factor genes showed differential expression patterns in response to drought, osmotic, and salt stresses, with the exception of *MeETR2*, *MeERF3*, and *MeERF14* which had transcript levels that were too low to detect (Fig 6, S7, S14–S16 Tables). *MeERS4* at 88.24% of the time points and *MeERF5* at 82.35% of the time points were induced in expression by drought, osmotic and salt stresses, but *MeERF10* and *MeERF11* at 94.12% of the time points, *MeERF12* at 100% of the time points, and *MeERF13* at 82.35% of the time points were suppressed by the three stresses (Fig 6). In general, the number of ethylene receptor genes and transcription factor genes that were induced was lower than the number of genes that were suppressed under all stress conditions (23.53% vs. 48.24%, respectively; S16 Table). Drought stress significantly induced the greatest number of ethylene receptor genes and transcription factor genes (34.17%) in cassava roots, followed by osmotic stress (18.00%) and salt stress (17.50%, S16 Table). The ranking for transcriptional suppression was salt stress (54.17%), osmotic stress (51.00%), and drought stress (40.00%, S16 Table).

The transcript levels of *MeERS2*, *MeERS3*, *MeERS4*, *MeERS5*, *MeEIN4*, *MeERF1*, *MeERF2*, *MeERF7*, *MeEIL1*, and *MeEIL2* were significantly up-regulated or unchanged compared to the control after drought stress treatment in cassava roots, except for *MeERS2* (3 d), *MeERS3* (6 d), *MeERF1* (9 d and 18 d), *MeERF2* (9 d), and *MeERF7* (9 d), which were significantly down-regulated at one or two time points. The expression levels of *MeERS1*, *MeERF5*, *MeERF6*, *MeERF8*, *MeERF9*, *MeERF10*, *MeERF11*, *MeERF12*, and *MeERF13* were significantly down-regulated or unchanged by drought stress treatment, except for *MeERS1* (18 d), *MeERF5* (15 d), *MeERF6* (12d and 15 d), *MeERF8* (12 d), *MeERF9* (12 d), and *MeERF10* (12 d), which were up-regulated at one or two time points in cassava roots (Fig 6).

*MeERS2*, *MeERS3*, *MeERS4*, *MeERS5*, *MeEIN4*, and *MeEIL1* were significantly induced or maintained at a level similar to the control after osmotic stress treatment in cassava roots, except for *MeERS2*, which was significantly suppressed at one time point (2 h). The expression levels of *MeERF1*, *MeERF2*, *MeERF4*, *MeERF6*, *MeERF7*, *MeERF8*, *MeERF9*, *MeERF10*, *MeERF11*, *MeERF12*, *MeERF13*, and *MeEIL2* were significantly down-regulated or unchanged at all time points after osmotic stress treatment, except for *MeERF9*, which was significantly induced at one time point (8 h) (Fig 6).

*MeERS4*, *MeERS5*, *MeERF5*, *MeERF6*, *MeERF8*, and *MeEIL2* were significantly induced or unchanged compared to the control after salt stress treatment in cassava roots, except for *MeERF6* (1 h and 4 h) and *MeERF8* (1 h), which were significantly suppressed at one or two time points. The expression levels of *MeERS1*, *MeERS2*, *MeEIN4*, *MeERF1*, *MeERF2*, *MeERF4*, *MeERF7*, *MeERF9*, *MeERF10*, *MeERF11*, *MeERF12*, and *MeERF13* were significantly down-regulated or unchanged at all time points after salt stress treatment in cassava roots (Fig 6).

### 3.8 Expression profiles of ethylene receptor genes and transcription factor genes in response to dehydration stresses in tuberous roots

**Fig 7:**
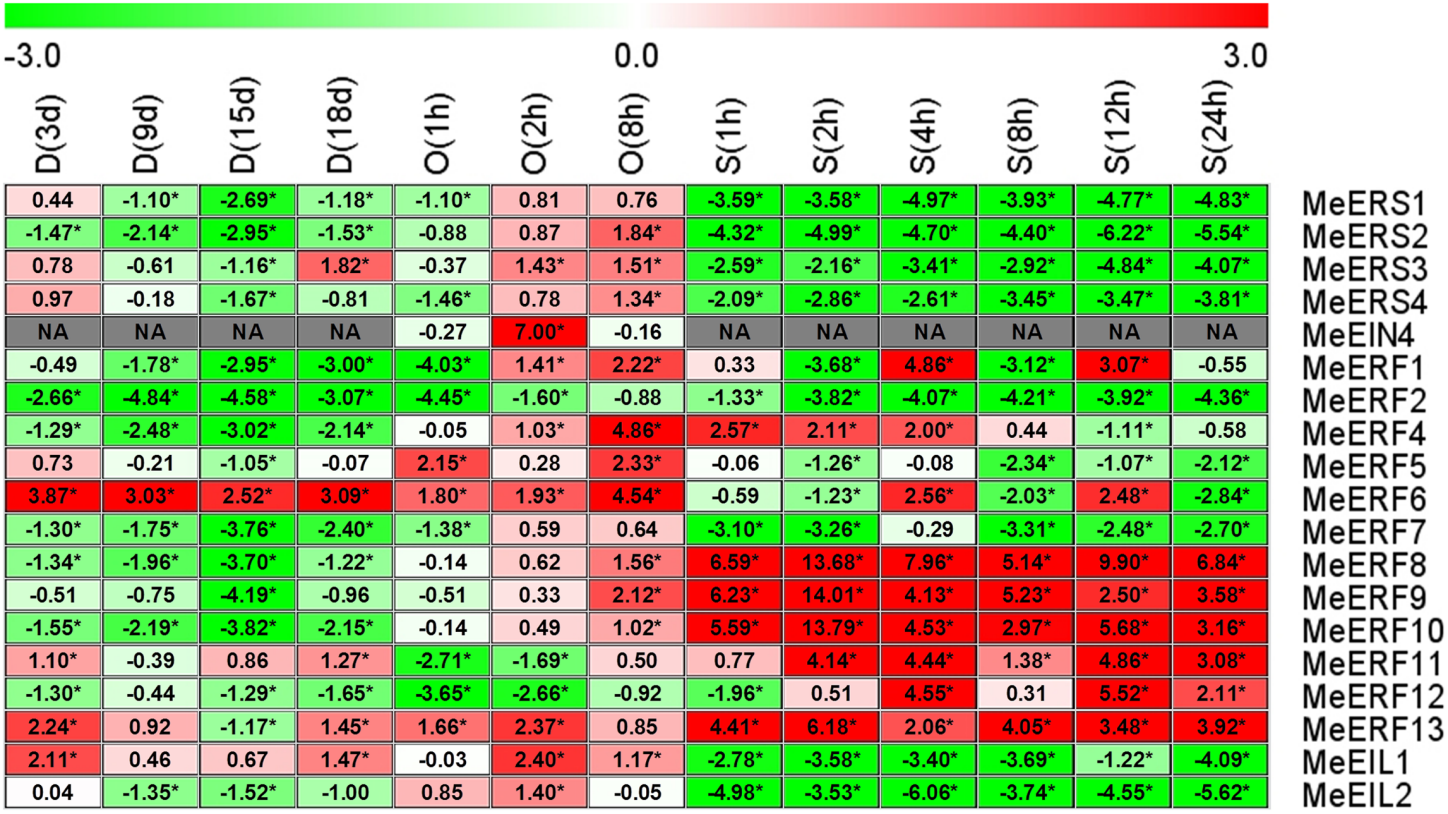
Expression profiles of ethylene receptor genes in cassava tuberous roots after drought, osmotic, or salt treatment. The threshold cycle (Ct) values of target and *MeActin* genes were obtained through qRT-PCR. The relative expression values for all selected genes was quantified using the 2^-ΔΔCt^ method, where ΔΔCt = (Ct _target gene_ − Ct _*MeActin* gene_) _treatment_ − (Ct _target gene_ − Ct _*MeActin* gene_) _control_ [37]. Ct of the formula represent mean Ct values of three repeats from three independent qRT-PCR assays. The heat map was generated based on the log_2_ (relative expression values) with MeV software. Red indicates high expression and white indicates low expression. D, drought stress; O, osmotic stress; S, salt stress. Asterisk (*) on the right corner of number indicate a significant difference at P < 0.05 (Student’s t-test) and absolute log_2_ (relative expression values) > 1 compared with non-treated control. NA denote the expression too weak to detect.

In tuberous root tissue, all of the tested ethylene receptor genes and transcription factor genes showed differential expression patterns in response to drought, osmotic and salt stresses, with the exception of some genes which had transcript levels that were too low for detection, including *MeERS5*, *MeETR2*, *MeERF3*, and *MeERF14* at all time points in response to all stresses (Fig 7, S7, S17–S19 Tables). *MeERF13* at 92.31% of the time points was induced in expression by drought, osmotic and salt stresses, but *MeERS2* at 84.62% of the time points, *MeERF2* at 100% of the time points and *MeERF7* at 84.62% of the time points were suppressed by the three stresses (Fig 7). In general, the number of induced ethylene receptor and transcription factor genes was clearly less than the number that were suppressed under all stress conditions in cassava tuberous roots (30.38% vs. 45.57%, respectively; S19 Table). The percentage of ethylene receptor genes and transcription factor genes induced by osmotic stress, salt stress, and drought stress were 38.60%, 36.11%, and 15.28%, respectively, in tuber, and the percentage of genes suppressed was 17.54%, 53.7%, and 55.56%, respectively (S19 Table).

The transcript levels of *MeERF6*, *MeERF11*, *MeERF13*, and *MeEIL1* were significantly up-regulated or unchanged after drought stress treatment in cassava tuberous roots, except for *MeERF13*, which was significantly down-regulated at 15 d. The expression levels of *MeERS1*, *MeERS2*, *MeERS4*, *MeERF1*, *MeERF2*, *MeERF4*, *MeERF5*, *MeERF7*, *MeERF8*, *MeERF9*, *MeERF10*, *MeERF12*, and *MeEIL2* were significantly down-regulated by drought stress treatment in cassava tuberous roots (Fig 7).

*MeERS2*, *MeERS3*, *MeEIN4*, *MeERF4*, *MeERF5*, *MeERF6*, *MeERF8*, *MeERF9*, *MeERF*10, *MeERF13*, *MeEIL1*, and *MeEIL2* were significantly induced or unchanged after osmotic stress treatment in cassava tuberous roots. The expression levels of *MeERS1*, *MeERF2*, *MeERF11*, and *MeERF12* were significantly down-regulated or unchanged at all time points after osmotic stress treatment in cassava tuberous roots (Fig 7).

*MeERF4*, *MeERF8*, *MeERF9*, *MeERF10*, *MeERF11*, *MeERF12*, and *MeERF13* were significantly induced after salt stress treatment in tuberous roots, except for *MeERF4*, which was significantly suppressed at 12 h and *MeERF*12 at 1 h. The expression levels of *MeERS1*, *MeERS2*, *MeERS3*, *MeERS4*, *MeERF2*, *MeERF5*, *MeERF6*, *MeERF7*, *MeEIL1*, and *MeEIL2* were significantly down-regulated at all time points after salt stress treatment in cassava tuberous roots, except for *MeERF6*, which was significantly suppressed at 4 h and 12 h (Fig 7).

### 3.9 Measurements of physiological indicators of plant stress

**Fig 8:**
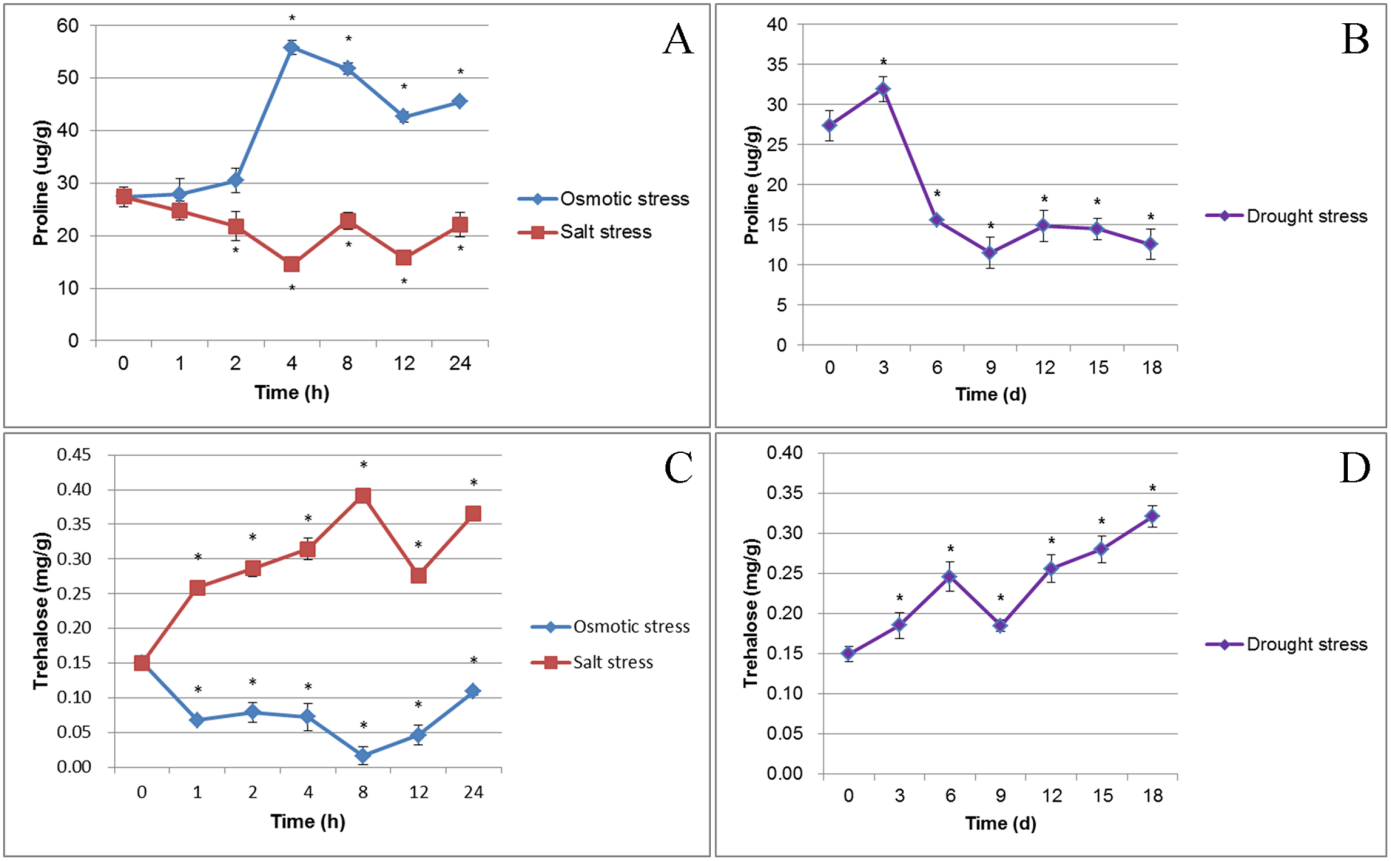
Trehalose and proline concentrations in cassava fresh leaves under drought, osmotic, or salt stress condition. Y-axes denote the concentration (error bars indicate standard deviation of three repeats from three independent assays, p < 0.05). A: The concentration of proline after salt and osmotic treatment; B: The concentration of proline after drought treatment; C: The concentration of trehalose after salt and osmotic treatment; D: The concentration of trehalose after drought treatment.

In this study, we also measured the trehalose and proline contents in cassava fresh leaves after drought, osmotic, and salt stress treatments (Fig 8, S20 and S21 Tables). In general, the trehalose and proline contents significantly changed in response to the three stress treatments. The levels of proline in response to osmotic and drought stress treatments exhibited the same patterns as trehalose levels in response to salt and drought stress treatment (an initial increase followed by a decrease and then an increase). Moreover, proline levels decreased at the final stages of drought stress treatment. The levels of proline in response to salt stress treatment exhibited the same patterns as the levels of trehalose in response to osmotic stress treatment (an initial decrease followed by an increase, decrease, and a final increase at end of treatment). At the 8 h time point, trehalose concentrations were highest under salt stress conditions and least abundant under osmotic stress conditions. In contrast to trehalose, proline showed a maximum accumulation at 4 h under osmotic stress conditions and minimum accumulation at 4 h under salt stress conditions and at 9 d under drought stress conditions (Fig 8, S20 and S21 Tables).

## 4. Discussion

Cassava is a perennial dicotyledonous crop native to tropical America [1, 2] that is widely cultivated for its tuberous roots, which are the principle source of calories for many of the world’s poorest people [3]. Cassava has the ability to produce tubers under poor growing conditions, such as low nutrients and drought [2, 4, 6]. However, the mechanisms underlying drought tolerance in cassava remain unclear. Ethylene as a gaseous hormone, is an endogenous regulator involved in plant responses to abiotic and biotic stresses [7, 8]. Although genes involved in ethylene transduction pathways play important roles in plant responses to stresses, they have not been fully investigated in cassava. In this study, we focused on genome-wide identification and molecular characterization of ethylene receptors and transcriptional factors in cassava tissues under non-stress and abiotic stress conditions, which may provide new ideas for further functional characterization of those genes and genetic improvement of cassava.

### 4.1. Characterization of ethylene receptors and transcription factors in cassava

Increasing evidence has indicated that the ethylene signaling pathway from receptors to transcription factors are involved in biotic and abiotic stress responses. In banana, *ERS2*, *ERS3*, and *ERS4* expression is enhanced by high temperature and *EIL2*, *EIL3* and *EIL4* are involved in fruit ripening [13]. The best-characterized *ERFs* are *ERF1*, which is activated by diverse stimuli including abiotic stresses, pathogens and oxidative stress in Arabidopsis (*Arabidopsis thaliana*) [46] and wheat (*Triticum aestivum*) [28]. *ERF3* plays an important role in resistance responses to osmotic and oxidative stresses in tobacco (*Nicotiana tabacum*) [47]. Surprised as it is, transcription of *MeERF3* and *MeERF14* genes was completely shut down in different cassava tissues after various abiotic stress treatments (Figs 4–7), no matter how abundent *MeERF3* and *MeERF14* genes were transcipted under non-stress condition (Fig 3). So *MeERF3* and *MeERF14* genes could be more sensitive than other *MeERFs* to drought, osmotic or salt stresses in cassava, which indicated that they would have important functions under non-stress condition. In various cassava tissues, all of the other genes measured showed differential regulation by various environmental stresses. Phylogenetic analysis suggested that Arabidopsis and cassava ERF proteins are very similar to each other and clustered into groups A, B, C and D, and ethylene receptors and EILs are clustered into groups A and B. Ethylene receptors and transcription factors were divided into several distinct groups, implying that they in these groups might have derived from a common ancestor (Fig 1).

### 4.2 Expression differences of ethylene signaling pathway related genes among different tissues under non-stress condition

Most of ethylene receptor genes and transcription factor genes were expressed in various organs/tissues under non-stress condition (Fig 3). However, their expression exhibited clear differences among different tissues. In general, the expression levels of the majority of the genes were lower in root and tuber, and higher in the leaf and stem. However, we were not able to detect *MeERF14* expression in tuberous root. *MeERS3*, *MeERF6*, *MeERF9*, *MeERF11*, and *MeERF12* were expressed highest in root, and *MeERF8* and *MeERF10* were expressed highest in tuberous root. This suggests that *MeERS3*, *MeERF6*, *MeERF9*, *MeERF11*, and *MeERF12* may function in the root in roles pertaining to the absorption of nutrients and water. Better absorption of nutrients and water could contribute to better resistance or tolerance to drought. *MeERF8* and *MeERF10* may participate in starch formation, which is helpful to tuberous root formation and development. Generally, the expression differences among tissues may reflect the complexity of gene family functions.

### 4.3 Expression differences of ethylene signaling pathway related genes among different tissues under abiotic stress conditions

Plants alternatively employ “survival” or “growth” strategies to cope with mild and severe restrictions in water availability, respectively [48]. When plants encounter severe dehydration stress conditions, they concomitantly up-regulate the transcript levels of stress response and tolerance related genes, and down-regulate the expression of growth and development related genes. Phytohormones are well-known to act as growth regulators in response to numerous stresses [49,50]. Ethylene as a hormone is involved in abiotic and biotic stresses [7, 8], and the ethylene signaling pathway, from upstream receptors to the downstream transcription factors, plays an important role in environmental stress during plant growth and development [9–11]. In various cassava organs, ethylene signaling pathway related genes exhibit differential expression profiles in response to drought stress, osmotic stress, and salt stress (Figs 4–7). This result was similar to the results presented for muskmelon [15].

After drought stress treatment, ethylene receptor genes and transcription factor genes were significantly induced in leaf, stem, root, and tuberous root at 47.62%, 37.93%, 34.17%, and 15.28% of all time points, respectively, whereas they were suppressed in tuberous root, root, stem, and leaf at 55.56%, 40.00%, 32.76%, and 19.84% of all time points, respectively (S22 Table). These results indicate that drought exposure triggered differential levels of ethylene signaling with the performance levels of the four cassava tissues as follows (from high to low): leaf, stem, root, and tuberous root. Our data suggest that leaf had the greatest response to drought stress via the ethylene signaling pathway, and tuberous root had the weakest response to drought stress in cassava. These results suggest that leaf has the greatest susceptibility to drought and tuberous root has the greatest tolerance. In order to reduce water loss by transpiration and conserve water under drought stress, cassava partially close their stomata of leaves and reduce leaf area by restricting the formation of new leaves, producing leaves of a smaller size, drooping leaves, and allowing leaves to fall [51–53]. Therefore, leaf formation is an important indicator used to assess drought tolerance in cassava [54]. However, reduction in leaf area during drought stress can lead to a reduction in the crop growth rate and yield, which is more pronounced in the shoots than in the roots and tuberous roots [54].

With regard to osmotic stress, ethylene receptor genes and transcription factor genes were significantly induced in tuberous root, leaf, stem, and root at 38.60%, 34.13%, 26.67%, and 18.00% of all time points, respectively, whereas they were suppressed in root, stem, tuberous root, and leaf at 51.00%, 45%, 17.54%, and 16.67% of all time points, respectively (S23 Table). Response levels to osmotic stress via the ethylene signaling pathway in four cassava tissues were as follows (high to low): tuberous root, leaf, stem, and root. After salt stress treatment, ethylene receptor genes and transcription factor genes were significantly induced in tuberous root, stem, leaf and root at 36.11%, 32.50%, 27.78%, and 17.50% of all time points, respectively, whereas they were suppressed in root, tuberous root, leaf, and stem at 54.17%, 53.70%, 30.95% and 30.83% of all time points, respectively (S24 Table). Response levels to salt stress via the ethylene signaling pathway in four cassava tissues were as follows (high to low): tuberous root, stem, leaf, and root. Our data suggest that tuberous root has the greatest response to osmotic and salt stresses, and root had the weakest response to osmotic and salt stresses through the ethylene signaling pathway in cassava. Surprisingly, tuberous root had the greatest response to osmotic and salt stress, but the weakest tissue response to drought tolerance. It is hypothesized that PEG-6000 and NaCl not only cause dehydration in cassava, similar to drought stress, but also disturb other physiological and metabolic processes of cassava. The key effect of salinity on cassava is a reduction in biomass, leaf area, and photosynthetic rate [55]. Severe concentrations (80–136.8 mM) of salt, which threaten the survival of most cassava varieties, increase water content of the roots of surviving cassava and induce water losses in the leaves but do not disturb the water content of the stem [56]. The biomass production of surviving plants has been shown to be negatively affected by severe concentrations of salt, which decreases the dry weight of stem tissues more than leaf tissues [56]. In contrast, root biomass was not affected by severe concentrations of salt, but tuber initiation was prevented [55, 56]. The salt concentration in tuberous roots in cassava grown at moderate concentrations (40–68 mM) of NaCl, which is generally not a threat to reproductive and vegetative growth in most cassava varieties, was nearly 20 times higher than in control plants, with 15x, 2x, and 4x concentrations in stems, leaves, and roots compared to to control plants, respectively [55, 56]. PEG-6000 may affect the growth or survival of various tissues in cassava through a different mechanism from salt stress, though this remains to be elucidated.

Our results show that these plant tissues had differential expression levels of genes involved in ethylene signaling in response to the stresses tested. Moreover, after several gene duplication events, the differential expression pattern of homologous genes in response to abiotic and biotic stresses may imply their functional diversity as a mechanism for adapting to the environment.

### 4.4 Carbohydrate contents of cassava leaves under stress conditions

In this study, we found that trehalose and proline contents increased at early stage of drought stress in cassava leaves. This phenomenon has also been observed in potato [57]. Trehalose accumulates in rice to improve resistance to salt stress [58, 59]. Proline accumulates in a wide variety of plants, including soybean [60, 61] and Arabidopsis [62]. Evidence suggests that trehalose and proline function as compatible solutes and perform other functions to protect plant cells against abiotic stresses and they play a prominent role in plant adaptation to various stresses [57–63]. On the whole, the proline concentration of cassava leaves also increased in response to osmotic stress, but the concentration of trehalose decreased in this study (Fig 8, S25 Table). Trehalose concentration of cassava leaves increased after drought and salt stress treatments, but the content of proline decreased. Furthermore, the regulation patterns of contents of proline and trehalose in response to various dehydration stresses were differential, or even the opposite (Fig 8, S25 Table). So we think that plant may not employ all of the stress-resistant substances to adapt to stress environment, in the face of single abiotic stress. And not only that, plant will take different coping strategies to deal with different stresses, when stresses come.

## Supporting information

S1 Table Primers used for qRT-PCR. (XLSX)

S2 Table The ethylene receptor genes and transcription factor genes used to construct the phylogenetic trees. (XLSX)

S3 Table Putative cis-elements of more than 6 bp identified in ethylene receptor genes and transcription factor genes. (XLSX)

S4 Table Expression data (log_2_ (expression values + 1)) of the ethylene receptor genes and transcription factor genes in different tissues of cassava. (XLSX)

S5 Table Expression data of the ethylene receptor genes and transcription factor genes in different tissues of cassava. (XLSX)

S6 Table Statistical analysis (standard error of mean) of expression data of the ethylene receptor genes and transcription factor genes in different tissues of cassava. (XLSX)

S7 Table Expression data (log_2_ (expression values + 1)) of *MeERF3* and *MeERF14* in different tissues of cassava at all timepoints after drought, osmotic and salt treatments. (XLSX)

S8 Table Expression data (log_2_-based values) of the ethylene receptor genes and transcription factor genes after various abiotic stress treatments in cassava leaves. (XLSX)

S9 Table Statistical analysis (Student's t-test) of expression data (log_2_-based values) of the ethylene receptor genes and transcription factor genes after various abiotic stress treatments in cassava leaves (t(0.05,4) = 2.776). (XLSX)

S10 Table Numbers of up- or down-regulated members of ethylene receptor genes and transcription factor genes in expression after various abiotic stress treatments in cassava leaves. (XLSX)

S11 Table Expression data (log_2_-based values) of the ethylene receptor genes and transcription factor genes after various abiotic stress treatments in cassava stems. (XLSX)

S12 Table Statistical analysis (Student's t-test) of expression data (log_2_-based values) of the ethylene receptor genes and transcription factor genes after various abiotic stress treatments in cassava stems (t(0.05,4) = 2.776). (XLSX)

S13 Table Numbers of up-regulated or down-regulated members of ethylene receptor genes and transcription factor genes in expression after various abiotic stress treatments in cassava stems. (XLSX)

S14 Table Expression data (log_2_-based values) of the ethylene receptor genes and transcription factor genes after various abiotic stress treatments in cassava roots. (XLSX)

S15 Table Statistical analysis (Student's t-test) of expression data (log_2_-based values) of the ethylene receptor genes and transcription factor genes after various abiotic stress treatments in cassava roots (t(0.05,4) = 2.776). (XLSX)

S16 Table Numbers of up-regulated or down-regulated members of ethylene receptor genes and transcription factor genes in expression after various abiotic stress treatments in cassava roots. (XLSX)

S17 Table Expression data (log_2_-based values) of the ethylene receptor genes and transcription factor genes after various abiotic stress treatments in cassava tuberous roots. (XLSX)

S18 Table Statistical analysis (Student's t-test) of expression data (log_2_-based values) of the ethylene receptor genes and transcription factor genes after various abiotic stress treatments in cassava tuberous roots (t(0.05,4) = 2.776). (XLSX)

S19 Table Numbers of up- or down-regulated members of ethylene receptor genes and transcription factor genes in expression after various abiotic stress treatments in cassava tuberous roots. (XLSX)

S20 Table The contents of proline and trehalose after various abiotic stress treatments in cassava leaves. (XLSX)

S21 Table Statistical analysis (Student's t-test) of proline and trehalose contents after various abiotic stress treatments in cassava leaves (t(0.05,4) = 2.776). (XLSX)

S22 Table Numbers of significantly up- or down-regulated members of ethylene receptor genes and transcription factor genes in expression in various cassava tissues after drought stress treatment. (XLSX)

S23 Table Numbers of significantly up-regulated or down-regulated members of ethylene receptor genes and transcription factor genes in expression in various cassava tissues after osmotic stress treatment. (XLSX)

S24 Table Numbers of significantly up-regulated or down-regulated members of ethylene receptor genes and transcription factor genes in expression in various cassava tissues after salt stress treatment. (XLSX)

S25 Table The changes of proline and trehalose contents after various abiotic stress treatments in cassava leaves, compared to the control. (XLSX)
